# Offspring mass variation in tree swallows: A case of bet‐hedging?

**DOI:** 10.1002/ecs2.2607

**Published:** 2019-03-07

**Authors:** Philippine Gossieaux, Martin Leclerc, Joanie Van de Walle, Yoanna Poisson, Pauline Toni, Julie Landes, Audrey Bourret, Dany Garant, Fanie Pelletier, Marc Bélisle

**Affiliations:** ^1^ Département de Biologie Université de Sherbrooke 2500 Boulevard de l'Université Sherbrooke Quebec J1K 2R1 Canada; ^2^ Present address: Applied Conservation Science Lab Department of Geography University of Victoria P.O. Box 1700, STN CSC Victoria British Columbia V8W 2Y2 Canada; ^3^ Present address: Raincoast Conservation Foundation P.O. Box 2429 Sidney British Columbia V8L 3Y3 Canada

**Keywords:** birds, diversified bet‐hedging, intra‐brood mass variation, reproductive success

## Abstract

The evolution of reproductive strategies is affected by the ability of organisms to deal with future environmental conditions. When environments are temporally unpredictable, however, it is difficult to anticipate optimal offspring phenotype. Diversification of offspring phenotypes, a strategy called diversified bet‐hedging, may allow parents to maximize their fitness by reducing between‐year variation in reproductive success. The link between diversification of offspring phenotypes and individual reproductive success, however, has rarely been documented empirically. We used an eight‐year dataset (1215 broods, 870 females) on individually marked tree swallows (*Tachycineta bicolor*) to assess whether intra‐brood mass variation was compatible with a diversified bet‐hedging strategy. Intra‐brood mass variation was weakly, but significantly repeatable within females, suggesting consistent individual differences. Greater intra‐brood mass variation, however, was not associated with reduced between‐year variation in reproductive success or increased female reproductive success. Moreover, contrary to diversified bet‐hedging expectations, fledging success of large broods was greater when hatchlings had similar rather than variable masses. Our results suggest that intra‐brood mass variation may not result from diversified bet‐hedging, but rather from complex interactions between environmental, brood, and maternal characteristics.

## Introduction

When environmental conditions are predictable, natural selection should favor the evolution of a single, optimal reproductive strategy (Stearns 1992, Roff 2002). Natural environments, however, often vary drastically over time and space (Southwood 1977). Anticipation of future environmental conditions is thus difficult for most organisms (Dall et al. 2005). In this context, long‐term individual reproductive success can be optimized by reducing the variance in reproductive success between reproductive events, a strategy referred to as bet‐hedging (Philippi and Seger 1989). The bet‐hedging hypothesis suggests that in unpredictable environments, a reduction in between‐year variability in reproductive success, which maximizes geometric mean fitness, is selected over a strategy of high reproductive success during some years coupled with total failure during others (Gillespie 1977, Philippi and Seger 1989, Olofsson et al. 2009).

In polytocous species, optimal reproductive strategies imply the optimization of a trade‐off between offspring size and number when resources are limited (Stearns 1989, Rollinson and Hutchings 2013). In this context, individuals may use a conservative bet‐hedging strategy, by producing a few large offspring that would have high survival probabilities regardless of environmental conditions (Philippi and Seger 1989, Marshall et al. 2008). Alternatively, a diversified bet‐hedging strategy should result in individuals consistently producing offspring of various phenotypes, increasing the probability that at least some of them will express the phenotype best adapted to a given environment (Philippi and Seger 1989, Marshall et al. 2008). Diversified bet‐hedging has been suggested as a potential mechanism explaining the large intra‐brood (or litter) mass variation observed in the wild (Marshall et al. 2008 and references therein). Under adverse environmental conditions, only the largest young are likely to survive (Marshall et al. 2008). In contrast, under favorable environmental conditions, most offspring should survive, regardless of their mass. As a result, producing only a few large offspring under such conditions would reduce maternal reproductive success (Marshall et al. 2008).

Most previous studies addressed the expected fitness of bet‐hedging strategies through modeling or simulations (Crean and Marshall 2009, Olofsson et al. 2009). The few empirical investigations of the fitness outcomes of bet‐hedging strategies have mainly been conducted on plants and invertebrates (reviewed in Simons 2011). Different taxa, however, face different constraints. Thus, widening the investigation of such questions to species from more taxa would provide a broader view of bet‐hedging strategies. Some studies on Chordata suggested mechanisms to explain the adaptive value of diversified bet‐hedging (e.g., developmental instability: Simons and Johnston 1997; hatching asynchrony: Laaksonen 2004). Yet, few have tested if this hypothesis is supported by empirical data (Marshall et al. 2008, Crean and Marshall 2009, Starrfelt and Kokko 2012).

The bet‐hedging hypothesis is usually tested at the population level by comparing reproductive success of parents over one reproductive event with variability in offspring phenotype (Koops et al. 2003, Einum and Fleming 2004). However, a better test of this hypothesis for iteroparous species would compare some measure of fitness over several reproductive events at the individual level (sensu Philippi and Seger 1989) and should not be limited to the study of variation in offspring traits alone (Philippi and Seger 1989, Hopper et al. 2003). Such test requires long‐term data on intra‐brood (or litter) variability from longitudinal studies on marked individuals, but this information is difficult to collect for wild animals. Most studies of diversified bet‐hedging in animal populations used measures of fetus size from a single reproductive event per individual (Gamelon et al. 2013), or measures of egg mass or size collected over several years from unidentifiable parents (Koops et al. 2003, Einum and Fleming 2004). A direct comparison of offspring trait variation to long‐term parental fitness in wild vertebrate populations is necessary to adequately test whether diversified bet‐hedging can explain intra‐brood (or litter) variation in offspring phenotypes, such as body mass.

While attributing offspring mass variation to a maternal strategy, such as diversified bet‐hedging, is appealing, offspring mass variation within a brood (or litter) could also arise from constraints on maternal ability to allocate resources evenly (Einum and Fleming 2004). The ability to evenly allocate resources among offspring can be reduced by limited food resources and physiological constraints (Arnold 1991). Considering the wide heterogeneity in individual reproductive potential typically found in natural populations (van Noordwijk and de Jong 1986), a range of female strategies dependent on their ability to diversify offspring mass can be expected (Gamelon et al. 2013, Yeager and Gibbons 2013). For instance, in wild boar (*Sus scrofa*), Gamelon et al. (2013) identified a continuum of female reproductive tactics depending on their body mass. Heavy females produced more offspring of variable sizes (diversified bet‐hedging), but light females produced fewer offspring of similar size (conservative bet‐hedging). To investigate the potential for diversified bet‐hedging, it is thus important not only to account for these potential sources of variation and their interactions, but also to evaluate them as alternative hypotheses for the observed variation in offspring mass.

Migratory birds face substantial environmental variation and unpredictability and cannot foresee what environmental conditions their offspring will face (Lack 1968, Newton 1998). As a migratory species, the tree swallow (*Tachycineta bicolor*) is subject to this environmental variation and unpredictability, both within and among breeding seasons (Dunn and Winkler 1999, Winkler et al. 2013, Bourret et al. 2015). Tree swallows are aerial insectivorous passerines associated with open habitats such as those found in agro‐ecosystems. They migrate from tropical wintering grounds to breed in early spring in temperate environments (Winkler et al. 2011, Knight et al. 2018). Their migratory behavior, combined with the large spatio‐temporal changes in food availability linked to agricultural landscapes (Rioux Paquette et al. 2013), suggests that tree swallows breeding in those landscapes cannot predict the environmental conditions in which offspring will develop. Indeed, agricultural landscapes are potential ecological traps (Gates and Gysel 1978), as human activities can cause a mismatch between habitat preference and fitness (Porlier et al. 2009, Baeta et al. 2012, Rioux Paquette et al. 2013). Moreover, as income breeders, swallows rely on resources available during the breeding season to produce and raise their offspring (Winkler and Allen 1995, Winkler et al. 2011). Therefore, they are highly vulnerable to cold spells (Winkler et al. 2013) and inter‐annual variations in environmental conditions (Baeta et al. 2012). In the face of this environmental unpredictability and variability, diversified bet‐hedging might allow female tree swallows to minimize variance in reproductive success among years.

We analyzed eight years of data from a longitudinal study on tree swallows to assess whether intra‐brood mass variation was compatible with the expectations from a diversified bet‐hedging strategy. First, we evaluated whether females showed repeatability in intra‐brood mass variation over several reproductive events. Intra‐brood mass variation must be repeatable within females to support a diversified bet‐hedging reproductive strategy. We also investigated alternative hypotheses to explain intra‐brood mass variation by including in our analyses other factors expected to affect offspring mass and female reproductive success, such as maternal characteristics (age and mass de Forest and Gaston 1996), brood size and clutch initiation date (Siikamäki 1998, Dunn and Winkler 2010), and environmental conditions (Erikstad et al. 1998, Siikamäki 1998). Second, we examined whether intra‐brood mass variation within a reproductive season conferred a fitness advantage by evaluating its relationship with the annual number of offspring fledged per female. Assuming that female tree swallows use a diversified bet‐hedging strategy with regard to intra‐brood mass, we predicted that the number of offspring fledged for a given year (our proxy of fitness) would be higher for females with large intra‐brood mass variations. Third, for females followed over multiple (≥2) reproductive events, we investigated the relationship between the geometric mean of intra‐brood mass variation and the geometric mean number of nestlings fledged. In line with a diversified bet‐hedging strategy, we predicted a positive correlation between the geometric mean of intra‐brood mass variation and the geometric mean number of nestlings fledged. Alternatively, an absence of relationship would suggest that intra‐brood mass variation may be explained by maternal and/or environmental factors rather than by a diversified bet‐hedging.

## Methods

### Species, study area, and data collection

Tree swallows winter in the southern United States, Mexico, and Central America and migrate to Canada and the northern United States to breed from April to August (Winkler et al. 2011). They can lay one to nine eggs, but most clutches range from four to seven eggs (Ghilain and Bélisle 2008, Winkler et al. 2011). Tree swallows are mainly singled‐brooded in the northern part of their breeding range, including our study area (Ghilain and Bélisle 2008, Winkler et al. 2011). Incubation lasts about 9–17 d (Coe et al. 2015), and nestlings receive 18–22 d of biparental care before fledging (Winkler et al. 2011). In our study area, mean fledging probability of hatchlings is 77% (Ghilain and Bélisle 2008, Bourret et al. 2017) and annual survival of adults varies between 30% and 60% (Butler 1988, Lagrange et al. 2014). Recapture probability of adult females in our study system is imperfect (0.86 ± 0.09 across years Lagrange et al. 2014) as females can either die, emigrate, or be unobserved. Therefore, we do not have complete life histories of all females, and to quantify long‐term reproductive success, we only considered females monitored over at least two reproductive events. Considering that tree swallow females start reproducing as one‐year‐olds (Winkler et al. 2011) and that only 6.2% of one‐year‐olds survive to the age of four (Butler 1988), we considered females monitored over at least two reproductive events to calculate an approximation for lifetime reproductive success.

Data were collected during the breeding seasons 2007–2014 in southern Québec, Canada. The study area covered 10,200 km^2^ and included a network of 400 nest boxes equally distributed among 40 farms. For more details on the study system, see Ghilain and Bélisle (2008). Each nest box was visited every two days, from nest building until the last nestling fledged, to monitor breeding activity. Information collected includes nest box occupancy (i.e., at least one egg laid), clutch initiation date of the first egg, clutch and brood sizes, and number of fledglings. During incubation, females were captured and weighed with a digital balance (±0.01 g). Newly observed tree swallows were ringed with a U.S. Fish and Wildlife Service aluminum band for individual identification. Females were aged as second year (SY, the year after their birth) or after second year (ASY) based on plumage (Hussell 1983). After the first recorded hatching (day 0), nestlings were counted and weighed at days 2, 6, 12, and 16. Nestlings were temporarily marked with a nail clipping code before being ringed with an aluminum band at 12 d. All animal handling procedures were approved by the Université de Sherbrooke Animal Care Committee, affiliated with the Canadian Council on Animal Care (protocol MB2014‐01).

### Data handling and description of the variables

Our analyses only considered the first breeding attempt by a female in a given year. In our study area, second clutches are only laid after complete hatchling failure of the first clutch. Second broods represent <5% of all broods for females monitored at least twice. Excluding second clutches allows comparisons to be made at similar temporal scales and between females having comparable energy reserves, hence avoiding the potential for results to be confounded by changes due to season and/or energetic loss associated with prior investment in first brood attempts.

We used the total number of fledglings per year per farm as a proxy of environmental conditions, where a low number of fledglings indicates poor environmental conditions as in Lagrange et al. (2017). We included this variable in the first statistical analysis to explain intra‐brood mass variation, but not in the second analysis to avoid circularity with the response variable (number of nestlings fledged; see *Statistical analyses* below).

Testing diversified bet‐hedging requires a test of the variation of a trait within a clutch. We thus calculated the coefficient of variation in nestling mass within a brood at day 2 (hereafter referred to as intra‐brood mass variation). As the coefficient of variation is known to be affected by sample size (Sokal and Rohlf 1995), we used the unbiased coefficient of variation as defined by Sokal and Rohlf (1981):

$$\text{CV}=\left(1+\frac{1}{4n}\right)\times \text{SD}\times \frac{100}{\overset{¯}{x}}$$

where *n* is the sample size (here the number of nestlings within a brood), SD is the standard deviation, and $\overset{¯}{x}$ is the mean (here the mean of nestling mass within a brood). We used mass on day 2 to capture the effect of early maternal tactics and to limit the potential noise in nestling mass that could be due to other factors, such as post‐hatching differential maternal allocation (Clutton‐Brock 1991).

For every female that produced nestlings at least twice (see Appendix S1: Fig. S1 for the distribution of the number of reproductive events per female), we also calculated the geometric mean intra‐brood mass variation across all reproductive events as well as the geometric mean reproductive success defined as the number of nestlings fledged. We used the geometric mean rather than the arithmetic mean due to its sensitivity to the variance and because previous empirical work showed that only the geometric mean responds to selection (Graham et al. 2014). Therefore, we estimated fitness by calculating the geometric mean reproductive success (μ) as:

$$\mu =\varpi -\frac{\sigma^{2}}{2\varpi }$$

where ϖ is the arithmetic mean of reproductive success and σ^2^ is the variance in reproductive success (according to Lacey et al. 1983).

### Statistical analyses

#### Intra‐brood mass variation

To quantify the influence of explanatory variables on intra‐brood mass variation, we developed 11 hierarchical candidate models including different combinations of blocks of variables that included maternal characteristics (female age class [SY or ASY] and body mass), brood characteristics (clutch initiation date [standardized per year per farm], brood size), and environmental conditions estimated as the total number of nestlings fledged per year per farm, where a low number of nestlings fledged indicates poor environmental conditions as in Lagrange et al. (2017), as well as two‐way interactions between brood size and all other variables, and between environmental conditions and all other variables (Appendix S1: Table S1). Summary statistics of all continuous variables used in this analysis can be found in Appendix S1: Table S2. To account for temporal changes in female mass, we controlled for the time elapsed between the onset of egg laying and measurement date, as well as for time of day when the measure was taken (Rioux Paquette et al. 2014). We fitted linear mixed‐effects models with the lme4 R package (Bates et al. 2015). We assessed the random‐effects structure with likelihood ratio tests, using the most complex fixed‐effects structure (model 11). Random effects included female identity, year, and farm. Once the random‐effects structure was selected, Akaike's information criterion, corrected for sample sizes (AIC_c_) was used to determine the most parsimonious model (Hurvich and Tsai 1989, Burnham and Anderson 2002). We square‐root‐transformed intra‐brood mass variation (see Appendix S1: Fig. S2 for the distribution of raw and square‐root‐transformed intra‐brood mass variation) to fulfill all statistical assumptions. We scaled (mean = 0, variance = 1) all independent variables to facilitate model convergence and interpretation of effect sizes. Finally, we used the best model to estimate the repeatability of intra‐brood mass variation using the rptR R package (Stoffel et al. 2017).

#### Short‐term reproductive success

To identify the factors affecting the number of nestlings fledged by a female in a given year, we developed five hierarchical candidate models including different combinations of blocks of variables that included intra‐brood mass variation, maternal characteristics, brood characteristics, and their two‐way interactions (Appendix S1: Table S3). We used generalized linear mixed‐effects models with a negative binomial error distribution implemented in the glmmADMB R package (Skaug et al. 2011). As above, we first selected the random‐effects structure using likelihood ratio tests and then used AIC_c_ to select the best combination of fixed effects. For both statistical analyses, all continuous variables were scaled (mean = 0, variance = 1) and were not multicollinear (VIF <2; Graham 2003).

#### Proxy of lifetime reproductive success

To test whether females that successfully reproduced at least twice in our study system behaved as predicted by a diversified bet‐hedging strategy, we calculated the Spearman rank correlation between the geometric mean of intra‐brood mass variation and the geometric mean number of nestlings fledged. All statistical analyses were performed in R 3.3.2 (R Core Team 2016).

## Results

### Intra‐brood mass variation

**Figure 1:**
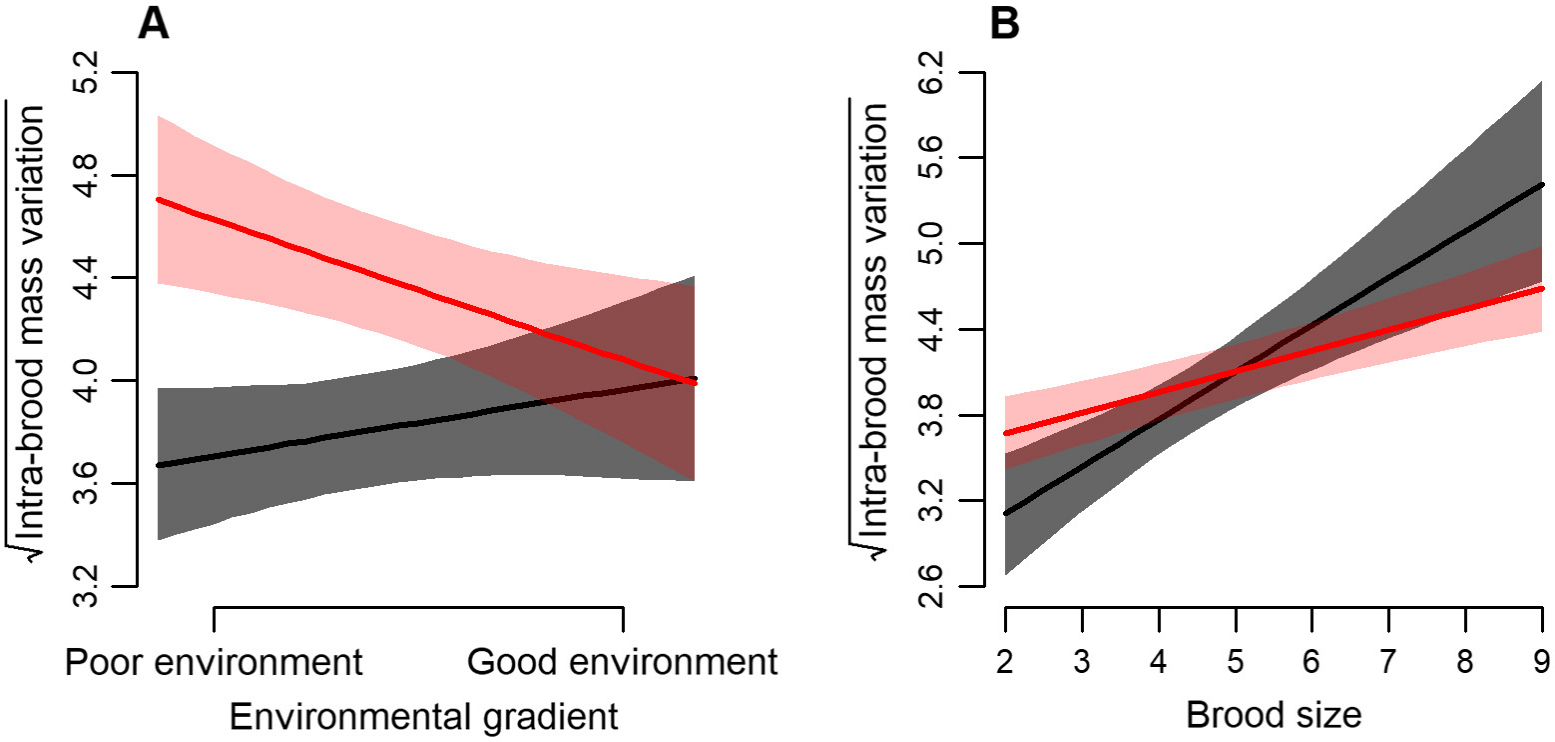
Relationships between intra‐brood mass variation (square‐rooted) and (A) environmental conditions depending on brood size (brood size of 3 in black and 7 in red) and (B) brood size depending on female age class (second year female in black and after second year female in red) for tree swallows in southern Québec, Canada, 2007–2014. The proxy of environmental condition is the number of offspring fledged per year per farm. Predictions of models are represented with their 95% confidence interval.

Between 2007 and 2014, we collected data from 1215 broods and 870 females. Most females (75%) nested only once in our study system (*n* = 649), but 220 females nested between two and seven times (mean ± SD = 2.56 ± 0.91; see Appendix S1: Fig. S2). The best random‐effects structure to explain intra‐brood mass variation in our candidate models included female identity (χ^2^ = 5.76, df = 1, *P *=* *0.017) and year (χ^2^ = 49.42, df = 1, *P *<* *0.001). The full model was the most parsimonious among 11 candidate models with an AIC_c_ weight of 0.60 (model 11; Appendix S1: Table S1). We found an interaction between the effects of brood size and environmental conditions on intra‐brood mass variation: Intra‐brood mass variation decreased with improving environmental conditions for larger broods but weakly increased with improving environmental conditions for smaller broods (Fig. 1A; Table 1). In contrast, there was a weak opposite trend for smaller broods. Intra‐brood mass variation increased with increasing brood size, and this effect was greater for younger females (SY; Fig. 1B). Intra‐brood mass variation was weakly repeatable across females (*n *=* *870, *r* = 0.11, 95% confidence interval = [0.03–0.21], *P *=* *0.008).

**Table 1:** Estimates (β), standard error (SE), and 95% CI of the fixed effects included in the most parsimonious model explaining intra‐brood mass variation of tree swallows in southern Québec, Canada, 2007–2014 (see Appendix S1: Table S1)

Fixed effects	β	SE	95% CI	
			Lower limit	Upper limit
**Intercept**	**4.094**	**0.094**	**3.909**	**4.279**
Female mass	0.055	0.035	−0.014	0.125
**Capture day**	**0.090**	**0.035**	**0.021**	**0.158**
Time of day	0.035	0.030	−0.024	0.094
Female age class–second year	0.049	0.090	−0.128	0.226
**Brood size**	**0.164**	**0.032**	**0.101**	**0.227**
Clutch initiation date	0.044	0.033	−0.021	0.109
Environment	−0.060	0.034	−0.127	0.007
**Environment × brood size**	−**0.060**	**0.029**	−**0.116**	−**0.004**
**Brood size × female age class–second year**	**0.189**	**0.092**	**0.009**	**0.369**
Brood size × clutch initiation date	0.035	0.030	−0.024	0.095
Environment × clutch initiation date	−0.062	0.033	−0.127	0.003
Environment × female age class–second year	0.052	0.084	−0.114	0.217

Notes

CI, confidence interval. All numerical variables were scaled. The dependent variable was square‐root‐transformed to fulfill all statistical assumptions. Coefficients for which 95% CIs exclude 0 are in bold.

### Short‐term reproductive success

**Figure 2:**
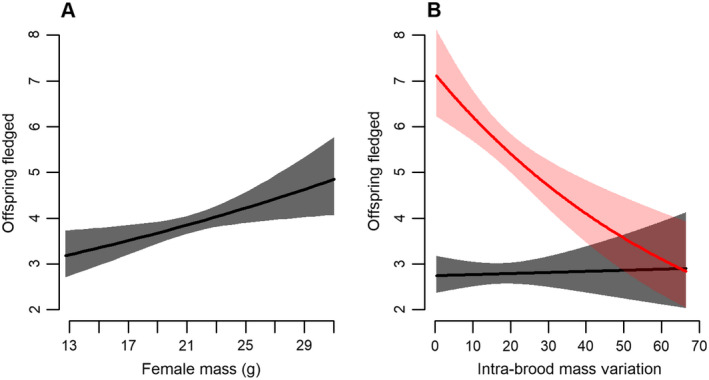
Relationships between the number of offspring fledged and (A) female mass and (B) intra‐brood mass variation depending on brood size (brood size of 3 in black and 7 in red) for female tree swallows in southern Québec, Canada, 2007–2014. Predictions of models are represented with their 95% confidence interval.

The full model was the most parsimonious with an AIC_c_ weight of 0.69 (Appendix S1: Table S3), with year (χ^2^ = 3.92, df = 1, *P* = 0.048) as a random effect. Female age and mass, brood size, and intra‐brood mass variation affected the number of nestlings fledged (Table 2). The number of nestlings that fledged was greater for heavier females (Fig. 2A). There was an interaction between brood size and intra‐brood mass variation: Intra‐brood mass variation had no effect on the number of nestlings fledged for smaller broods, but a strong negative effect for larger broods (Fig. 2B).

**Table 2:** Estimates (β), standard error (SE), and 95% CI of the fixed effects included in the most parsimonious model explaining the number of fledglings of female tree swallows in southern Québec, Canada, 2007–2014

Fixed effects	β	SE	95% CI	
			Lower limit	Upper limit
**Intercept**	**1.360**	**0.028**	**1.305**	**1.415**
**CVmass** a	−**0.056**	**0.018**	−**0.091**	−**0.021**
**Female mass**	**0.049**	**0.018**	**0.015**	**0.084**
Time of day	−0.004	0.016	−0.034	0.027
Capture day	−0.006	0.018	−0.042	0.030
**Female age class–second year**	−**0.099**	**0.047**	−**0.192**	−**0.006**
**Brood size**	**0.204**	**0.017**	**0.170**	**0.238**
Clutch initiation date	−0.011	0.017	−0.044	0.023
CVmass × clutch initiation date	−0.005	0.017	−0.038	0.027
CVmass × female age class–second year	−0.059	0.048	−0.152	0.035
**CVmass × brood size**	−**0.036**	**0.014**	−**0.063**	−**0.009**
Brood size × female age class–second year	0.098	0.054	−0.009	0.204
Brood size × clutch initiation date	0.003	0.018	−0.031	0.038

Notes

CI, confidence interval. All numerical variables were scaled. Coefficients for which 95% CIs exclude 0 are in bold.

a Intra‐brood mass variation calculated as the unbiased coefficient of variation in nestling mass within a brood.

### Proxy of lifetime reproductive success

**Figure 3:**
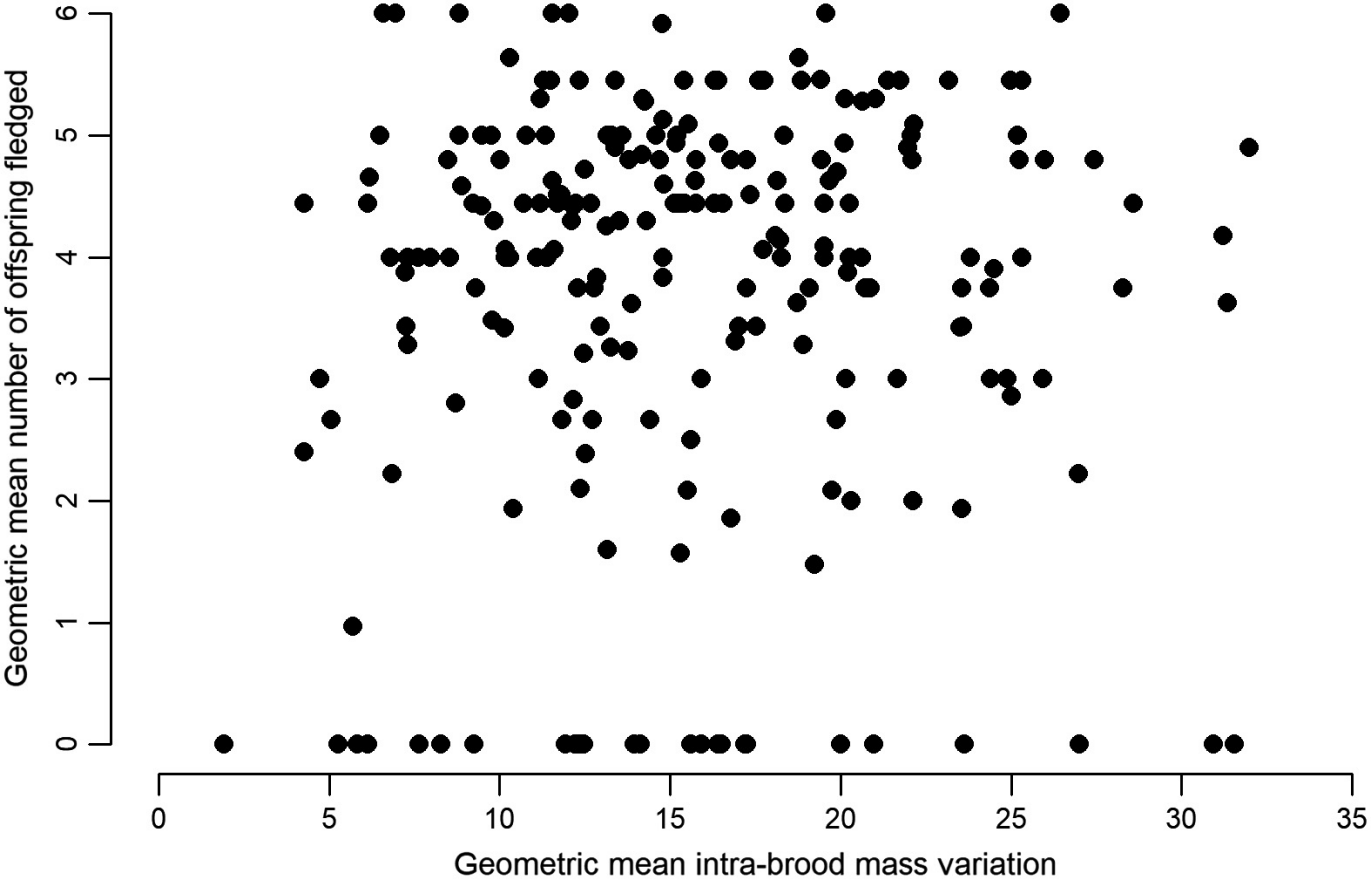
Relationship between the geometric mean intra‐brood mass variation and the geometric mean number of offspring fledged calculated over several reproductive events for tree swallows (each point represents one individual) in southern Québec, Canada, 2007–2014 (*r*_S_ = 0.004, *P *=* *0.95).

For 220 females that produced nestlings twice or more in our study system, the geometric mean of intra‐brood mass variation was not correlated with the geometric mean number of nestling fledged (*r*_S_ = 0.004; *P *=* *0.95; Fig. 3).

## Discussion

Our main objective was to evaluate whether intra‐brood mass variation may result from a diversified bet‐hedging strategy in female tree swallows under unpredictable environmental conditions. We showed that intra‐brood mass variation was slightly repeatable within females, but the main predictions of diversified bet‐hedging were not met. There was no correlation between intra‐brood mass variation and our proxy of lifetime reproductive success. We found, however, that intra‐brood mass variation was negatively associated with short‐term reproductive success for larger broods. Our results suggest that intra‐brood mass variation does not confer fitness advantages in tree swallows, but rather depends on complex interactions between environmental conditions, brood size, and female age.

Diversified bet‐hedging is often suggested as a mechanism explaining why females produce offspring of various phenotypes (Koops et al. 2003, Gamelon et al. 2013). However, diversified bet‐hedging, as first formally defined by Philippi and Seger (1989), requires that females gain a fitness advantage by diversifying offspring phenotypes. We found consistent differences in intra‐brood mass variation between females, but females that produced more variable broods did not gain a reproductive advantage, in terms of number of nestlings fledged when investigated over one or several reproductive events. In large broods, high intra‐brood mass variation was associated with poor fledging success, suggesting that rather than being adaptive, intra‐brood mass variation may be due to physiological or environmental constraints. This process was previously suggested in blue tits (*Cyanistes caeruleus*), where food‐supplemented females produced clutches with smaller egg‐mass variation than non‐supplemented ones, indicating that offspring mass variation was probably the result of energetic constraints (Nilsson and Svensson 1993).

It could be argued that the probability of detecting diversified bet‐hedging would have been higher if investigated at an earlier stage of reproduction, for example, by looking at intra‐brood variability in egg mass (Koops et al. 2003). Although we did not have data on egg mass, egg mass is correlated with hatchling mass in tree swallows (Whittingham et al. 2007). Also, despite the common use of fledging success as a fitness proxy for birds (Endler 1986, Weatherhead and Dufour 2000, Keedwell 2003), we cannot rule out the possibility that intra‐brood mass variation could be linked to other proxies of female fitness. For instance, since offspring mass affects post‐fledging traits such as survival (Naef‐Daenzer et al. 2001, Monrós et al. 2002, Cleasby et al. 2010) or migratory capacity (Merilä and Svensson 1997), intra‐brood mass variation could also affect these traits and therefore impact female fitness indirectly. However, data on post‐fledging survival and migratory behavior are not available for our study system.

Although we did not find any evidence for diversified bet‐hedging, intra‐brood mass variation across years was repeatable within females. Apart from a diversified bet‐hedging strategy, weak repeatability of intra‐brood mass variation may arise from other factors related to female identity. For instance, the positive relationship between female mass and reproductive success could suggest that females with greater energy supplies consistently produce more fledglings, as female mass positively affects clutch size and fledging success (see Fig. 2A; Winkler and Allen 1995, Rioux Paquette et al. 2014, Millet et al. 2015, Pellerin et al. 2016). Our repeatability analyses controlled for female mass, but several other factors linked to female identity could influence offspring mass and reproductive success, such as inter‐individual variation in ability to acquire and allocate resources to reproduction (van Noordwijk and de Jong 1986, Glazier 2000, Takahashi et al. 2003), and access to high‐quality resource patches (Pusey et al. 1997, Altmann and Alberts 2003). It should be noted, however, that while intra‐brood mass variation was significantly repeatable, female identity only explained 12% of this trait, suggesting that intra‐brood mass variation is mostly driven by factors other than maternal characteristics.

The strong relationship between intra‐brood mass variation and environmental conditions suggests that the alternative hypothesis of factors limiting female allocation decisions may explain intra‐brood mass variation better than a strategy of diversified bet‐hedging. Under poor environmental conditions, large broods showed greater variability in mass than small broods, likely reflecting a trade‐off between brood size and offspring mass (Smith and Fretwell 1974, Smith et al. 1989, Pellerin et al. 2016). For instance, a female producing only a few offspring may be able to allocate a large proportion of her energy to each of them, thereby reducing intra‐brood variation in mass. In contrast, a female producing a large brood may not be able to allocate resources evenly among nestlings and may thus be forced to bias her allocation to ensure that at least some of them will survive, increasing intra‐brood mass variation. This trade‐off seemed to strongly affect young females who may face higher energetic constraints than older females, which are usually more experienced (Rioux Paquette et al. 2014) and may be more efficient in energy acquisition and allocation (Robertson and Rendell 2001). While we found that larger broods are more variable in mass under unfavorable environmental conditions, which suggests an offspring quantity vs. quality trade‐off, this relationship vanished in good environments. This result is consistent with previous theoretical and empirical studies showing that life‐history trade‐offs are more apparent under unfavorable environmental conditions (van Noordwijk and de Jong 1986, Stearns 1992, Gillespie et al. 2008).

In our system, environmental conditions vary greatly within and between reproductive seasons and can have a large impact on within‐year reproductive success of tree swallows (Lessard et al. 2014, Rioux Paquette et al. 2014, Bourret et al. 2015, Millet et al. 2015). Indeed, a post hoc analysis showed that there is no temporal autocorrelation (Appendix S1: Figs. S3, S4) in our proxy of environmental condition across years, suggesting that females cannot predict environmental conditions based on experience. Other studies have suggested that diversified bet‐hedging is only likely to evolve under extreme cases of environmental unpredictability (McKee 1997, Einum and Fleming 2004, Dziminski et al. 2009). In our study system, environmental unpredictability may not be extreme enough to favor the emergence of diversified bet‐hedging. Our results therefore suggest that the observed intra‐brood phenotypic variation is more likely to be a consequence of environmental constraints limiting the females’ ability to feed their nestlings rather than a strategy to minimize between‐year fluctuations in reproductive success.

The ability of females to successfully rear offspring is constrained by the availability of resources, especially for income breeders in unpredictable environments. Our results advise against systematically interpreting phenotypic variation in offspring traits as a manifestation of diversified bet‐hedging. That variation can result from parental heterogeneity in reproductive potential and capacity to cope with environmental variations across years. Income breeders make allocation decisions based on available resources during the rearing period, which may explain the strong relationship between intra‐brood mass variation and local environmental conditions in tree swallows. Capital breeding may alleviate environmental constraints on the breeding grounds, providing females more flexibility in reproductive tactics.
