# Supplementary material for: Offspring mass variation in tree swallows: A case of bet‐hedging?
Source: Ecosphere. 2019 Mar 7;10(3):e02607. doi: 10.1002/ecs2.2607 (PMC9286465; doi:10.1002/ecs2.2607)
Supplement: Supplementary file 1 [file ECS2-10-e02607-s001.pdf]

Appendix S1

Journal: Ecosphere

Title of the article: Offspring mass variation in tree swallows: A case of bet-hedging?

Authors: Philippine Gossieaux, Martin Leclerc, Joanie Van de Walle, Yoanna Poisson, Pauline Toni, Julie Landes, Audrey Bourret, Dany Garant, Fanie Pelletier and Marc Bélisle

Table S1: Candidate models for the analyses on intra-brood mass variation

Table S2: Summary statistics of the variables used in the analyses

Table S3: Candidate models for the short-term reproductive success analyses

Figure S1: Graph of the number of reproductive events per female

Figure S2: Histograms of intra-brood mass variation

Figure S3: Temporal autocorrelation analyses

Figure S4: Temporal autocorrelation analyses

Table S1. Candidate linear mixed models assessing the influence of brood and maternal characteristics, as well as environmental conditions (total number of fledglings produced on a farm on a given year), on intra-brood mass variation (*n* = 1215) of tree swallows in southern Québec, Canada, 2007-2014. Female identity and year were included as random effects in all models. Candidate models are listed with their number of parameters (K), log likelihood (LL), the difference in corrected Akaike Information Criterion compared to the best-supported model (ΔAICc), and model weight (w_i). The model with the lowest AICc is specified in bold.

Model	Covariates	K	LL	ΔAICc	w_i
1	Female mass + capture day[†] + time of day[‡] + female age class	8	-1729.0	44.2	0.000
2	Brood size + clutch initiation date	6	-1713.3	8.7	0.008
3	Environment	5	-1735.3	50.8	0.000
4	Model 1 + Model 2	10	-1708.3	6.8	0.020
5	Model 1 + Model 3	9	-1728.9	46.0	0.000
6	Model 2 + Model 3	7	-1712.2	8.5	0.008
7	Model 2 + brood size × clutch initiation date	7	-1711.8	7.9	0.012
8	Model 1 + Model 7 + female age class × brood size	12	-1703.9	2.2	0.204
9	Model 3 + Model 7 + environment × brood size + environment × clutch initiation date + environment × female age class	11	-1705.4	3.2	0.121
10	Model 1 + Model 2 + Model 3	11	-1706.9	6.2	0.027
**11**	**Model 10 + female age class × brood size + brood size × environment + clutch initiation date × brood size + environment × clutch initiation date + environment × female age class**	**16**	**-1698.7**	**0.0**	**0.600**

[†] Capture day refers to the number of days between the first egg laid and the day female body mass was measured (see Methods for details).

[‡] Time of day is when female body mass was taken (see Methods for details).

Table S2. Mean, standard error (SD), minimum and maximum values for the continuous variables used in the models.

Variable	Mean	SD	Min	Max
CVmass	17.55	8.63	0.34	66.53
Brood size	4.90	1.14	2	9
Environment	22.83	11.57	0	53
Female mass	21.30	1.98	12.71	31.03
Capture day[†]	15.77	6.13	6	41
Time of day[‡]	0.52	0.11	0.30	0.91
Clutch initiation date	0	5.20	-18.75	32.71

[†] Capture day refers to the number of days between the first egg laid and the day female body mass was measured (see Methods for details).

[‡] Time of day refers to the time of the day female body mass was taken (see Methods for details).

Table S3. Candidate models assessing the influence of brood and maternal characteristics, as well as intra-brood mass variation (CVmass), on the number of fledglings of female tree swallows (*n* = 1,215) in southern Québec, Canada, 2007-2014. Year was included as a random effect in all models. Candidate models are listed with their number of parameters (K), log likelihood (LL), the difference in corrected Akaike Information Criterion compared to the best-supported model (ΔAICc), and model weight (w_i). The model with the lowest AICc is specified in bold.

Model	Covariates	K	LL	ΔAICc	w_i
1	CVmass	4	-2615.8	234.6	0.000
2	Female mass + capture day[†] + time of day[‡] + female age class + brood size + clutch initiation date	9	-2499.9	13.1	0.001
3	Model 2 + Brood size × female age class + brood size × clutch initiation date	11	-2498.7	14.6	0.000
4	Model 1 + Model 2	10	-2493.2	1.6	0.307
**5**	**Model 1 + Model 3 + CVmass × Clutch initiation date + CVmass × Female age class + CVmass × Brood size**	**15**	**-2487.3**	**0.0**	**0.691**

[†] Capture day refers to the number of days between the first egg laid and the day female body mass was measured (see Methods for details).

[‡] Time of day refers to the time of the day female body mass was taken (see Methods for details).

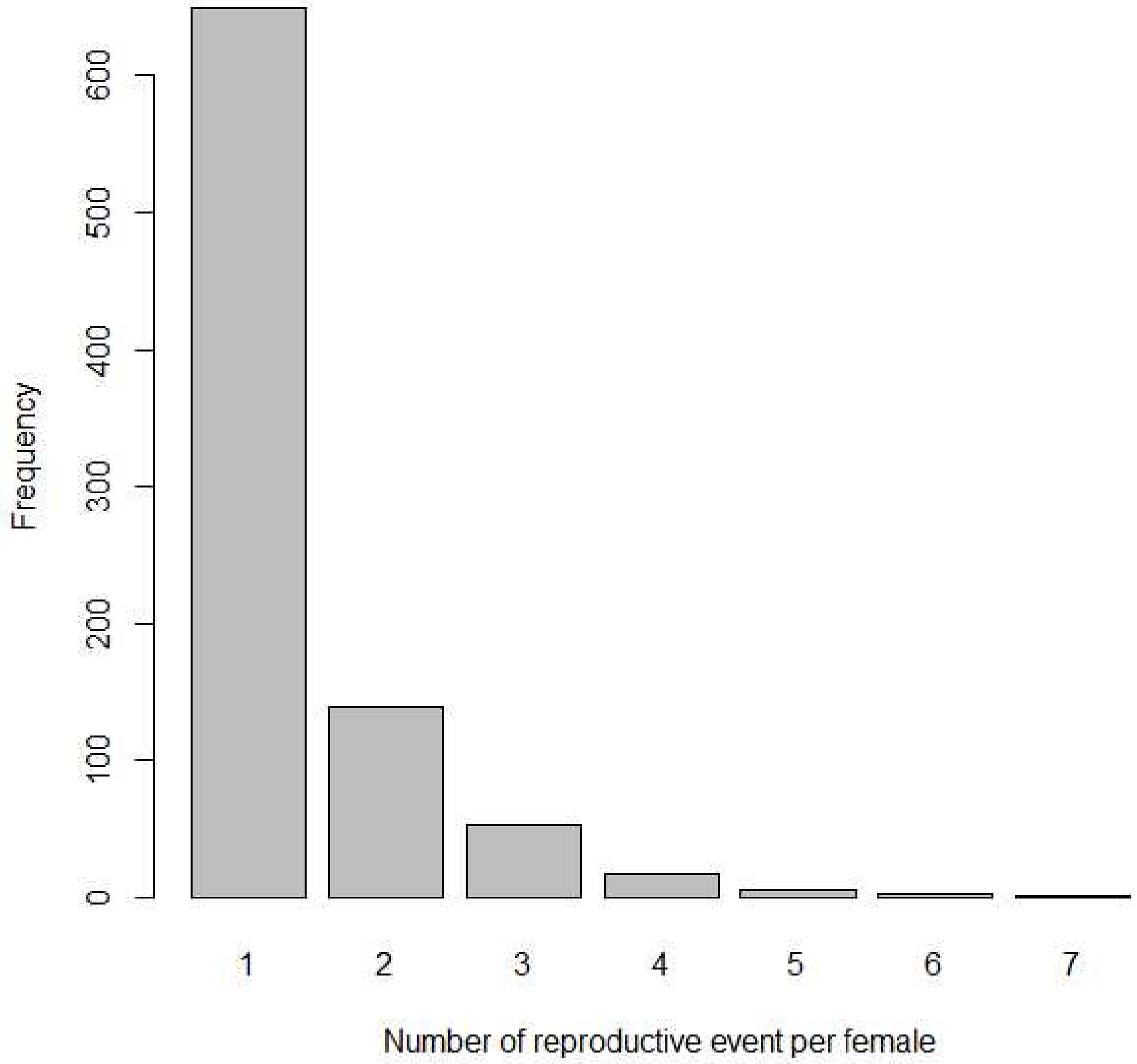

Figure S1 – Frequency of the number of reproductive events per female.

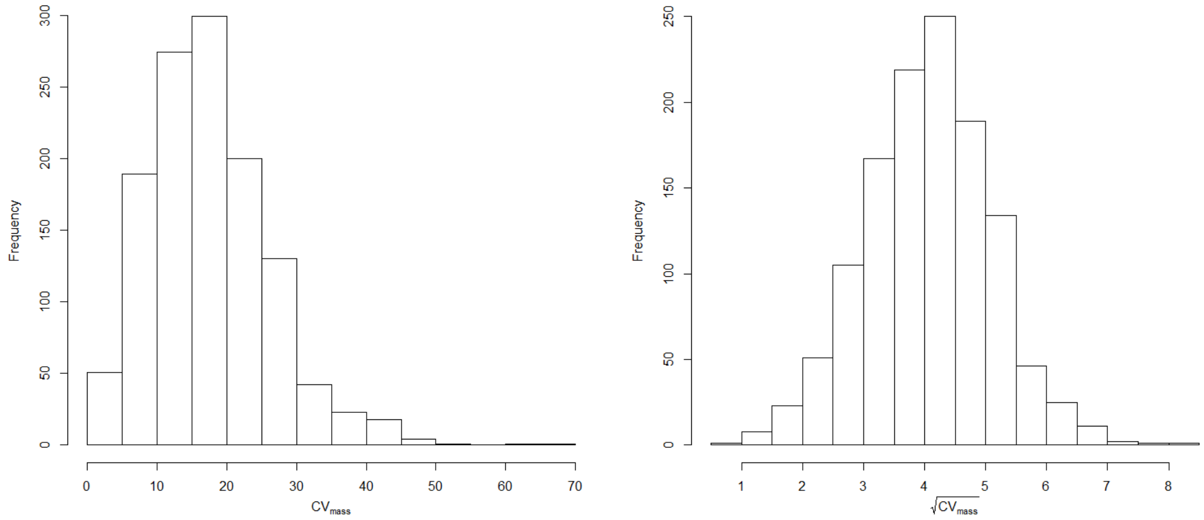

Figure S2 – Raw and square-root transformed intra-brood mass variation (*n* = 1215).

Temporal autocorrelation analysis

We evaluated if environmental condition (the number of fledglings) was temporally autocorrelated across years. We first ran an autocorrelation function on the total number of fledglings produced in our study system (Figure S3). We then ran an autocorrelation function on the total number of fledglings produced on each farm and pooled the results at each time lag (Figure S4). The results show no temporal autocorrelation in environmental condition.

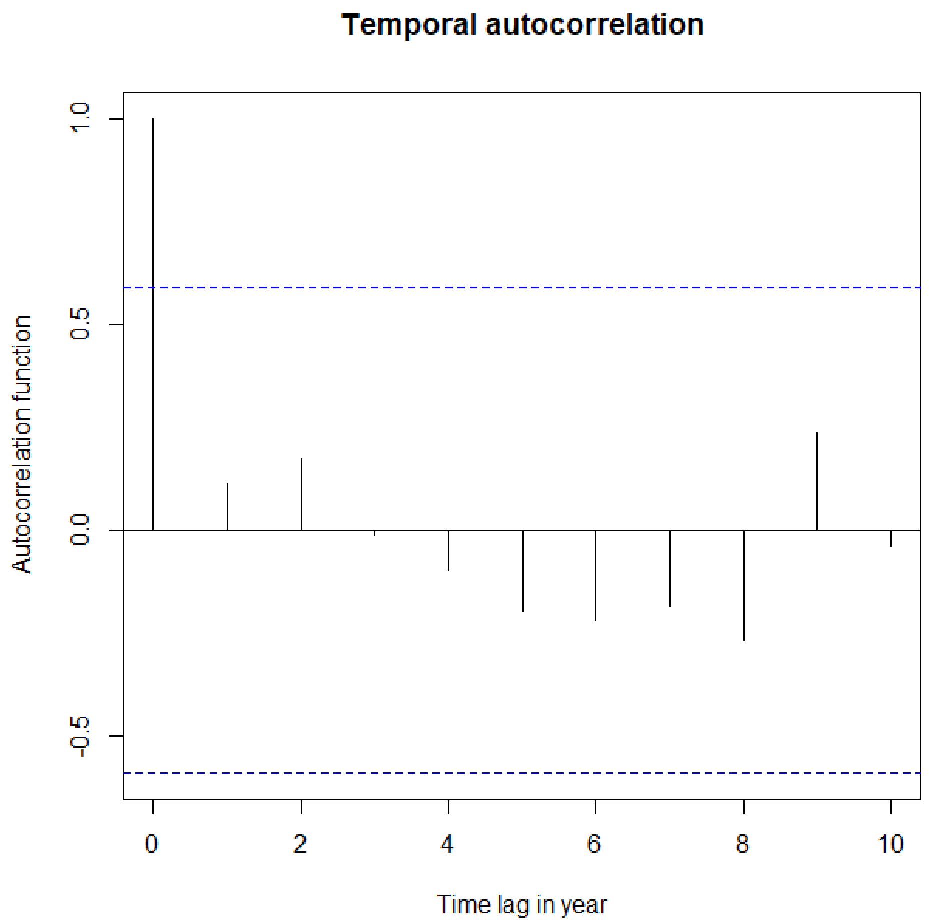

Figure S3 – Temporal autocorrelation in environmental condition in our study system. Dashed blue lines represent 95% confidence intervals. The results showed that there is no temporal autocorrelation in environmental condition.

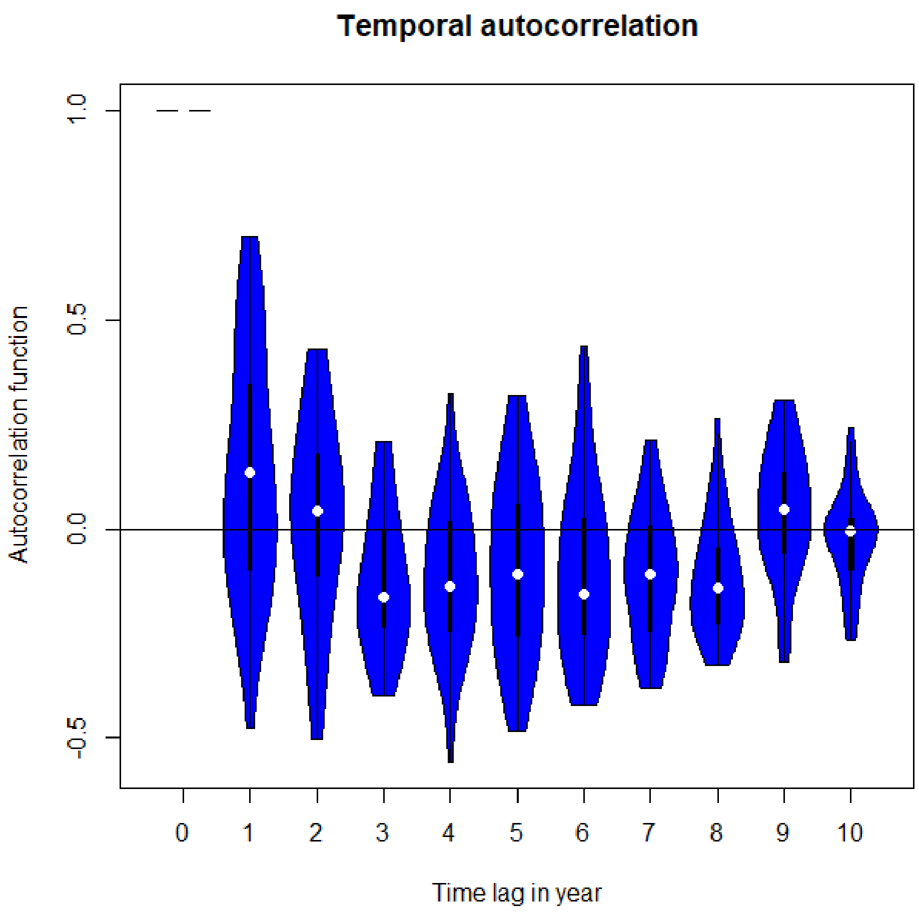

Figure S4 – Temporal autocorrelation in environmental condition in our study system. Autocorrelation functions were run on each farm separately and then pooled together at each time lag. There is no temporal autocorrelation in environmental condition.
